# Supplementary material for: An atlas of late prenatal human neurodevelopment resolved by single-nucleus transcriptomics
Source: Nat Commun. 2022 Dec 12;13:7671. doi: 10.1038/s41467-022-34975-2 (PMC9744747; doi:10.1038/s41467-022-34975-2)
Supplement: Supplementary file 1 — Supplementary Information [file 41467_2022_34975_MOESM1_ESM.pdf]

An atlas of late prenatal human neurodevelopment resolved by single-nucleus transcriptomics

Ramos et al

Supplementary Information

Supplementary Figure 1 (relates to Figure 1)

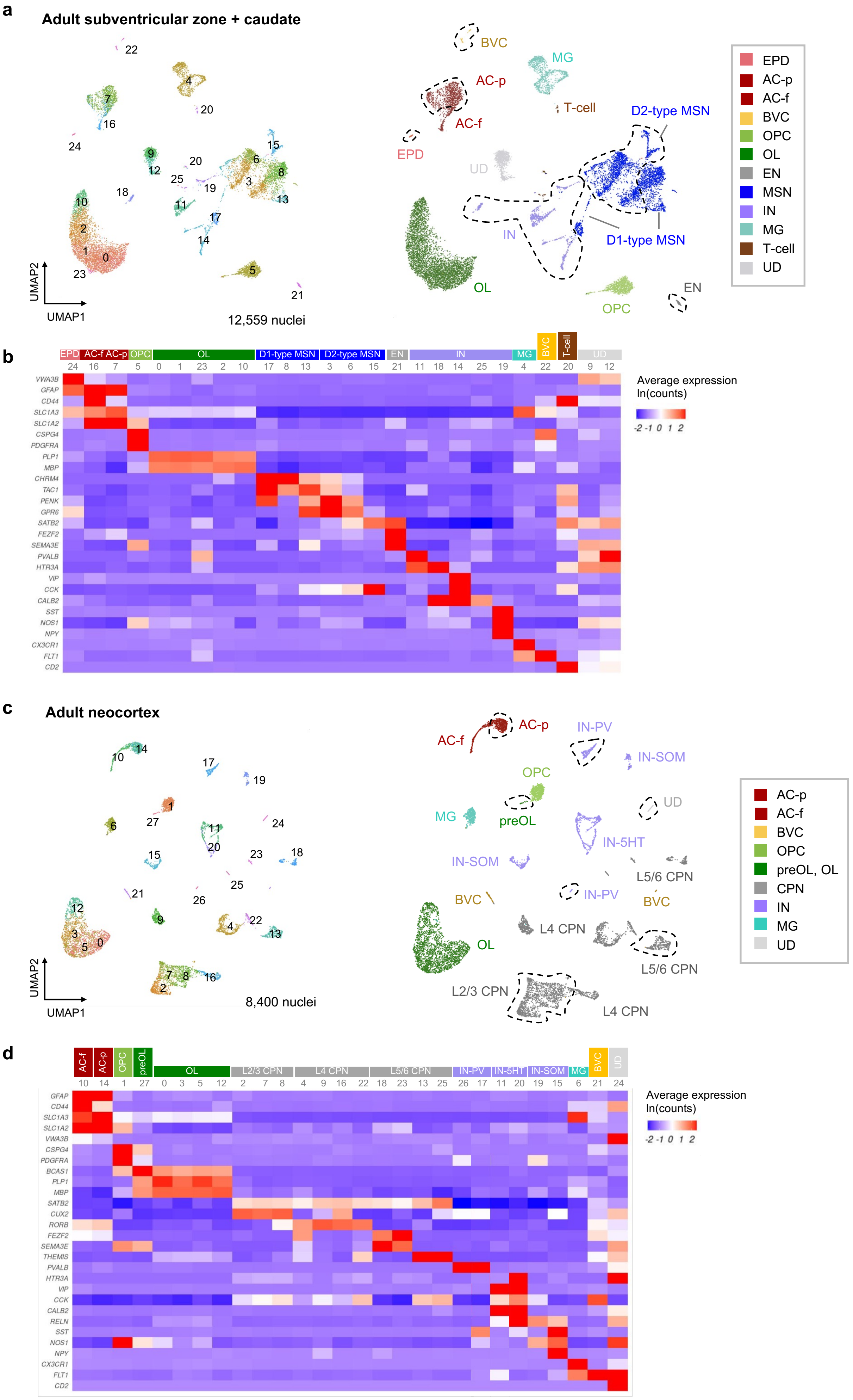

Supplementary Fig. 1 Transcriptomic atlas of adult subventricular zone and cortical plate a-b. UMAP plots (a) and heatmap of average log-normalized gene expression per cluster (b) derived from adult SVZ + caudate dissections, n=3 patients, annotated based on canonical cell-type markers. **c-d.** UMAP plots (c) and heatmap of average log-normalized gene expression per cluster (d) of adult neocortex dissections from same n=3 patients as in a-b, annotated based on canonical cell-type markers.

Supplementary Figure 2 (relates to Figure 1)

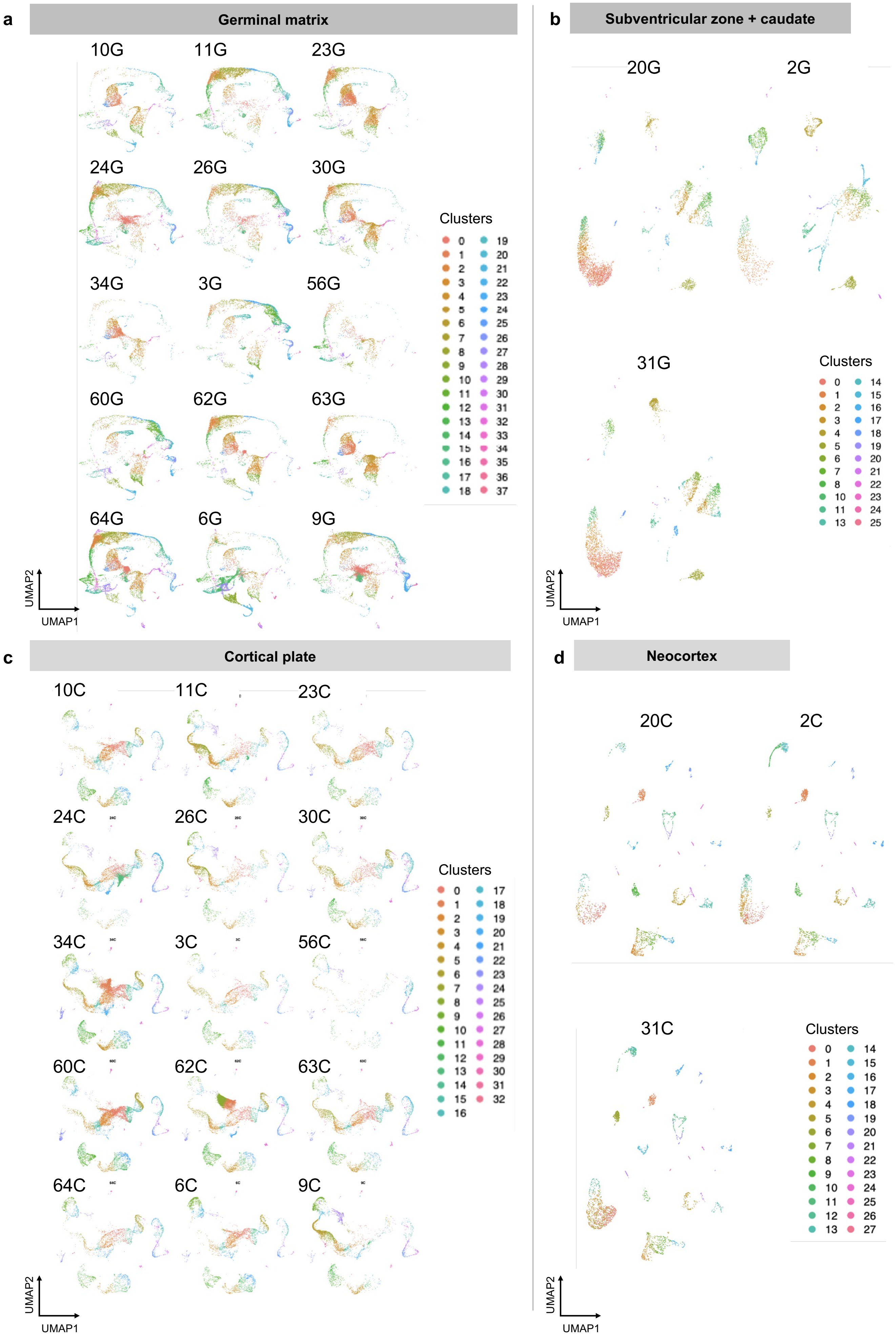

Supplementary Fig. 2 Individual sample contribution to integrated objects. **a-d.** Integrated UMAPs of germinal matrix (a), subventricular zone + caudate (b), cortical plate (c), and neocortex (d), showing each sample contributing to each cell-type cluster. Cells are colored by cluster annotation.

Supplementary Figure 3 (relates to Figure 1)

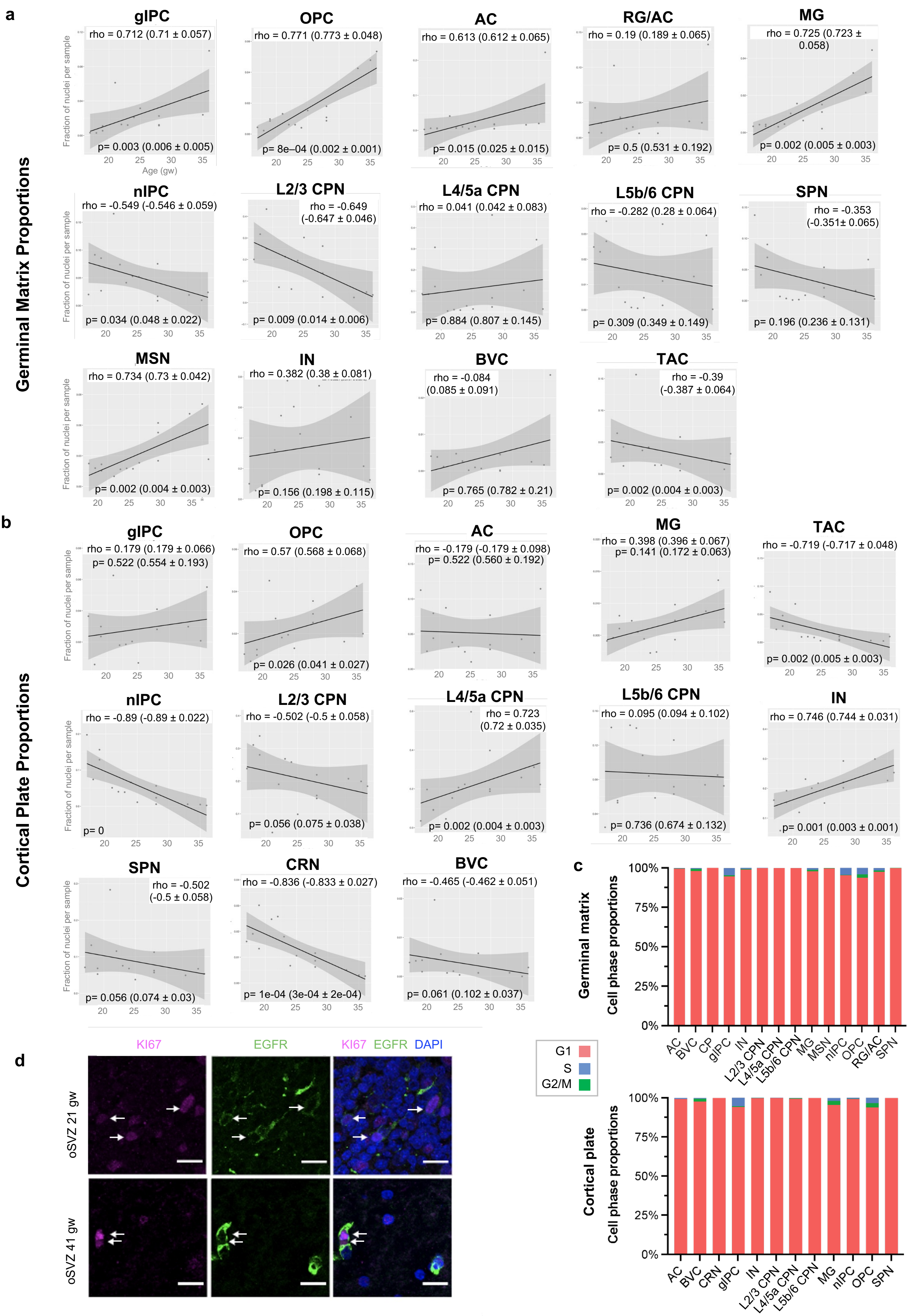

Supplementary Fig. 3 Cell type composition a. Germinal matrix cell type proportions per sample correlated to gestational age. **b.** Cortical plate cell type proportions per sample correlated to gestational age. The correlation coefficient (ρ / rho) and significance (p-value) in (a-b) are determined by two-tailed Spearman's rank-order correlation test. Confidence intervals are calculated by leave-one-out analysis. Error bands in (a-b) represent confidence intervals. Source data for (a-c) are provided as a Source Data file. **c.** Cell cycle phase analysis within the integrated prenatal germinal matrix (top) and cortical plate (bottom). Fraction of nuclei per cell type with high (>0.25) S and G2/M cell cycle score, indicative of a proliferative cell state. **d.** Immunohistochemical analysis for EGFR (green) and KI67 (pink). Co-localization in second (21 gw) and third (41 gw) trimester germinal matrix tissue samples is indicated by arrows. Immunofluorescence experiments were repeated three independent times, with similar results. Scale bars = 15μM.

Supplementary Figure 4 (relates to Figure 2)

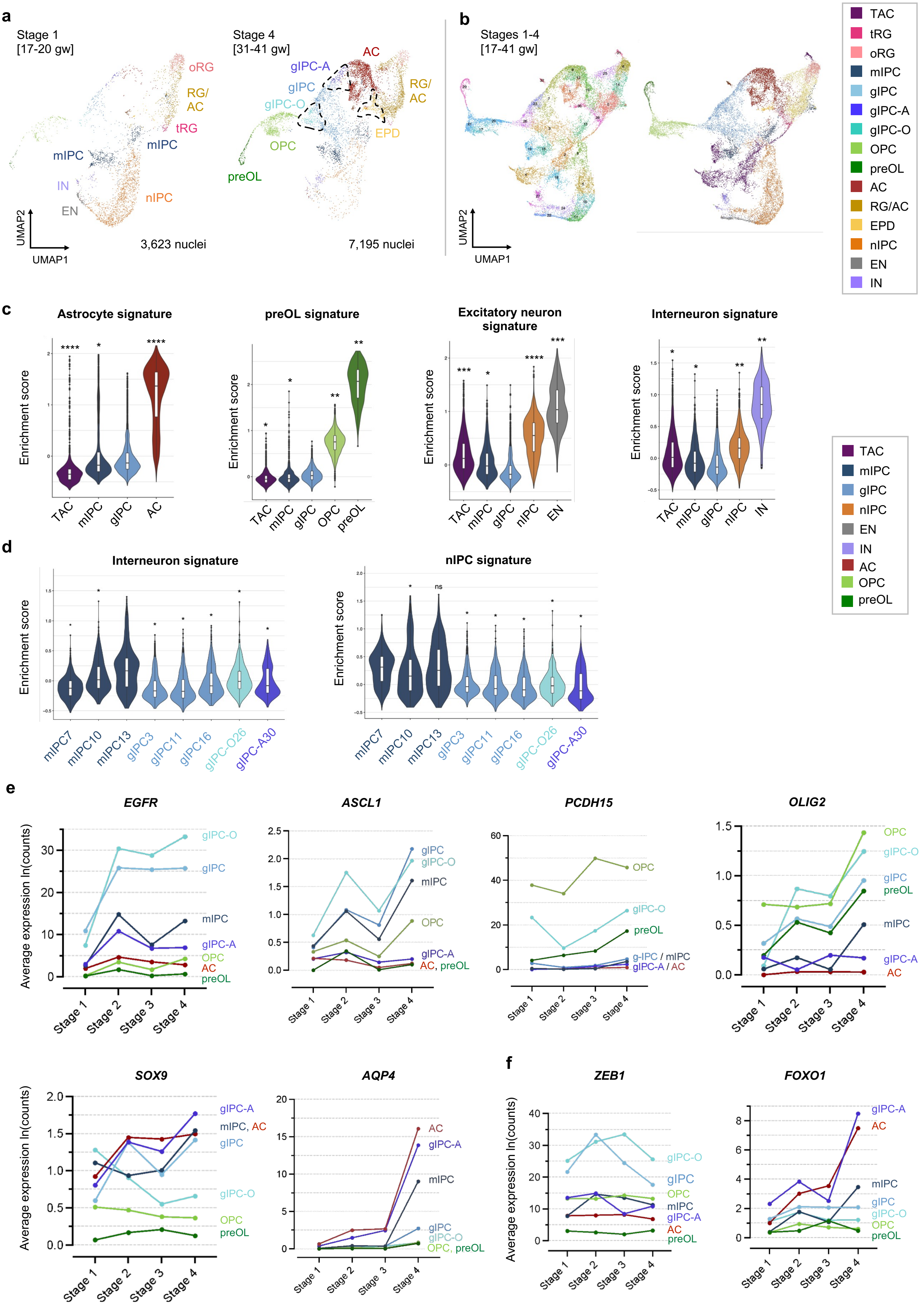

Supplementary Fig. 4 Stage-specific gene expression and lineage enrichment analyses a. Stage specific UMAPs of subclustered germinal matrix analysis in Fig. 2a. **b.** UMAP of subclustered GM analysis without cell cycle regression. **c.** Violin plots of maturation analysis using annotation in (b). **d.** Violin plots of neuronal enrichment analysis for mIPC and gIPC subpopulations, see also Fig. 2e-f. Scores in (c-d) are generated using top 50 DEGs for each respective GM cell type. Statistically significant differences between subclusters in (c-d) are determined using pairwise two-sided Wilcoxon Rank Sum tests with Holm correction (* $p < 1e-5$, ** $p < 1e-100$, *** $p < 1e-200$, and **** $p < 1e-300$), showing the significance between gIPC against all others in (c) and the significance between the highest scoring population against all others in (d). Box plots in (c-d) show the median, bounds of box represents the first and third quartile, and whiskers extend to the minimum and maximum values within 1.5 times of the interquartile range. Individual data points represent outliers. **e-f.** Line graphs of log-normalized average gene expression per stage, demonstrating dynamic changes in gIPC, astrocyte, and OPC marker expression (e) and in *FOXO1* (left) and *ZEB1* (right) expression (f), in glial subtypes. Source data for (c-i) are provided as a Source Data file.

Supplementary Figure 5 (relates to Figure 2)

a Germinal matrix cell type proportions (subclustered annotations)

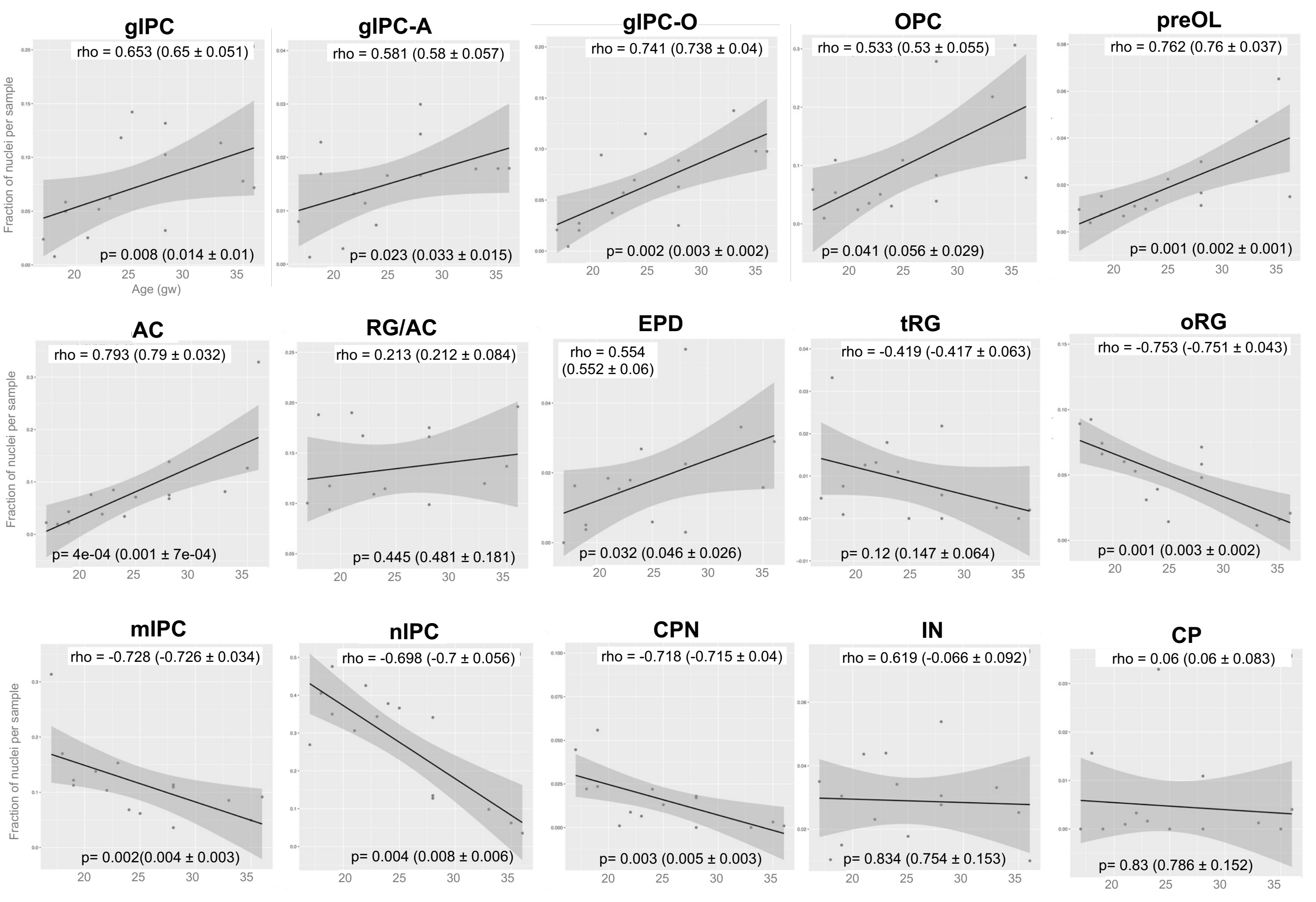

b Cortical plate cell type proportions (subclustered annotations)

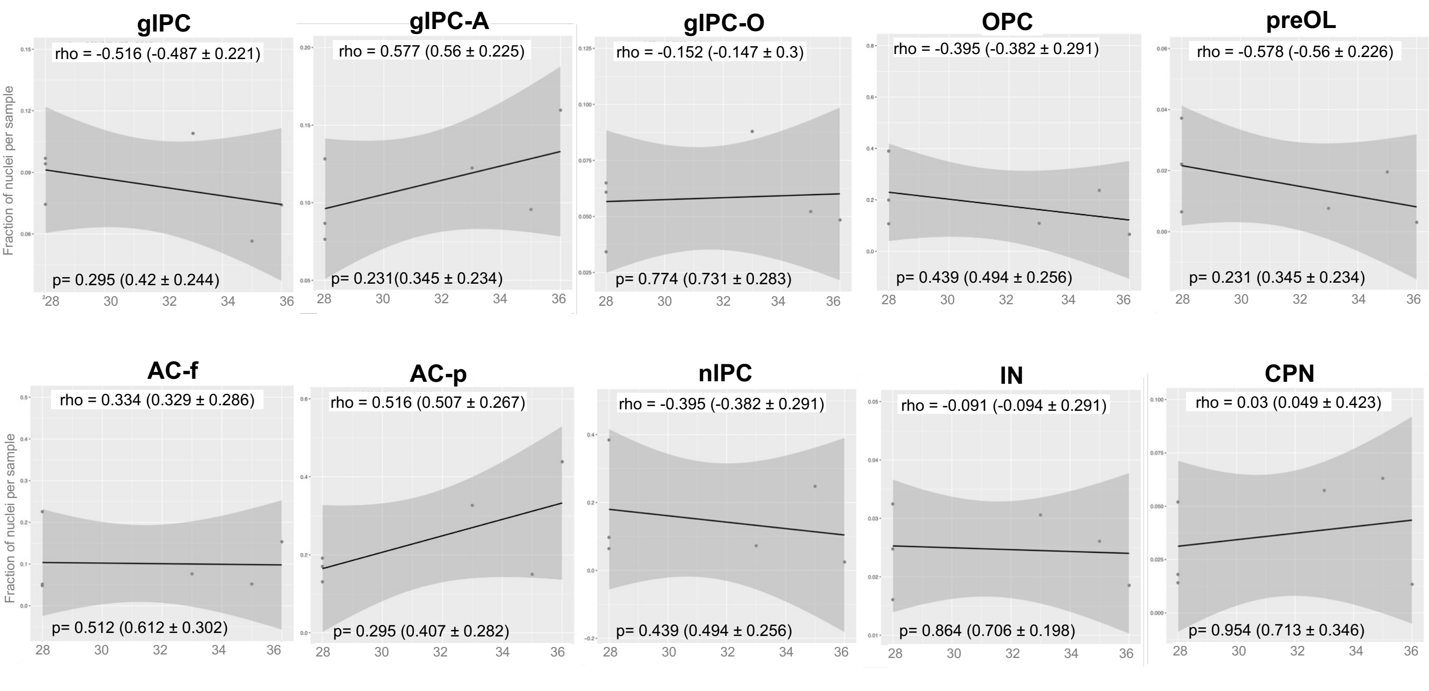

Supplementary Fig. 5 Cell type proportion analysis using subclustered annotations a. Subclustered germinal matrix cell type proportions per sample correlated to gestational age. **b.** Subclustered cortical plate cell type proportions per sample correlated to gestational age. The correlation coefficient (ρ / rho) and significance (p-value) in (a-b) are determined by two-tailed Spearman's rank-order correlation test. Confidence intervals are calculated by leave-one-out analysis. Error bands in (a-b) represent confidence interval. Source data are provided as a Source Data file.

Supplementary Figure 6 (relates to Figures 2 and 4)

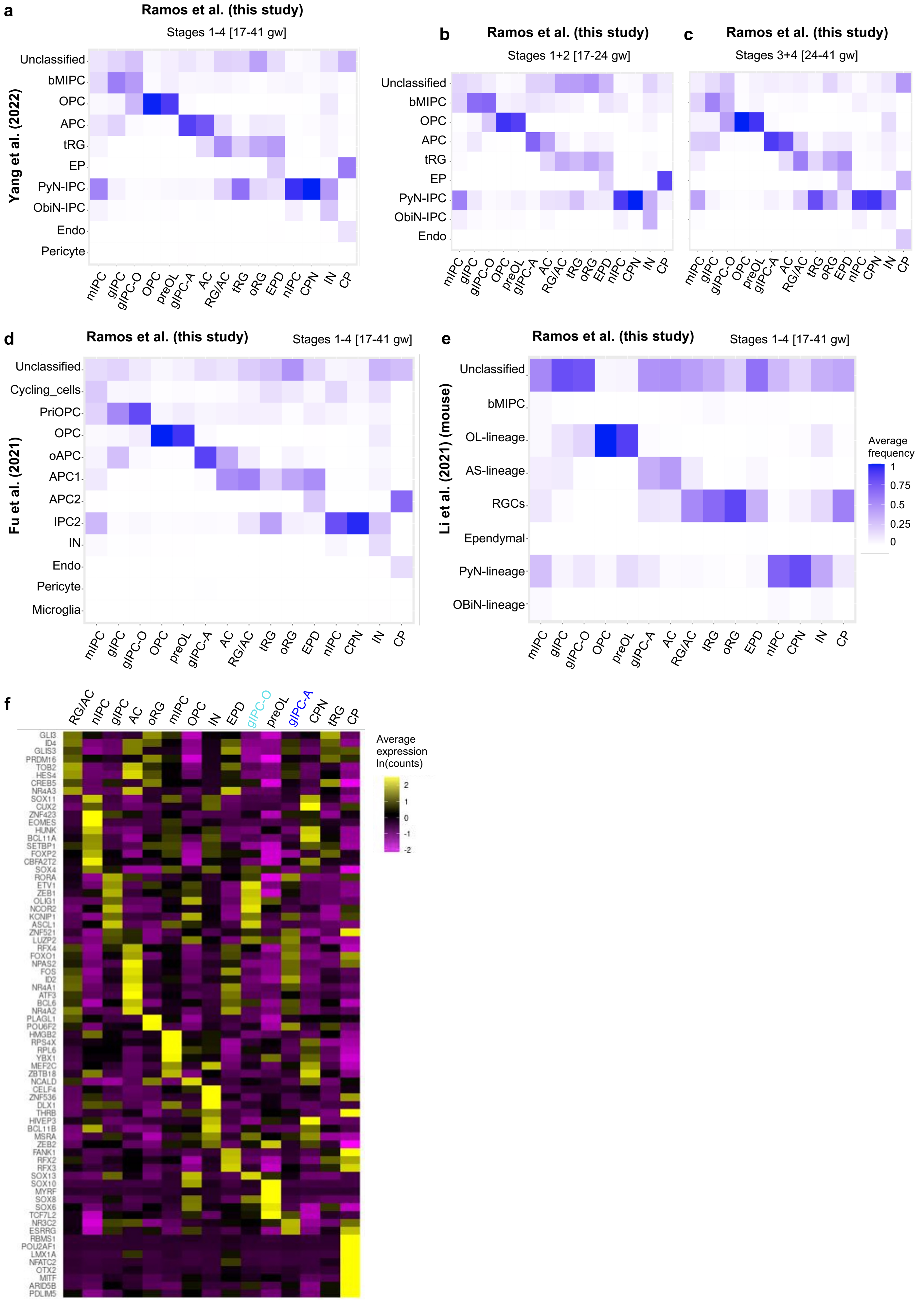

Supplementary Fig. 6 Correspondence analysis of germinal matrix annotations to published datasets a-e. Confusion matrix representation of frequency match between our subclustered GM annotations (Ramos et al.) and published developmental cell type annotations by Yang et al. (2022) in human (a-c), Fu et al. (2021) in human (d), and Li et al. (2021) in mouse (e)**.** Nuclei with maximum prediction scores of < 0.5 are marked as unclassified. Source data for (a-e) are provided as a Source Data file. **f.** Heatmap of log-normalized average gene expression per cell type identity (subclustered GM annotations) showing top differential TF marker expression for each cell subtype.

Supplementary Figure 7 (relates to Figures 5 and 6)

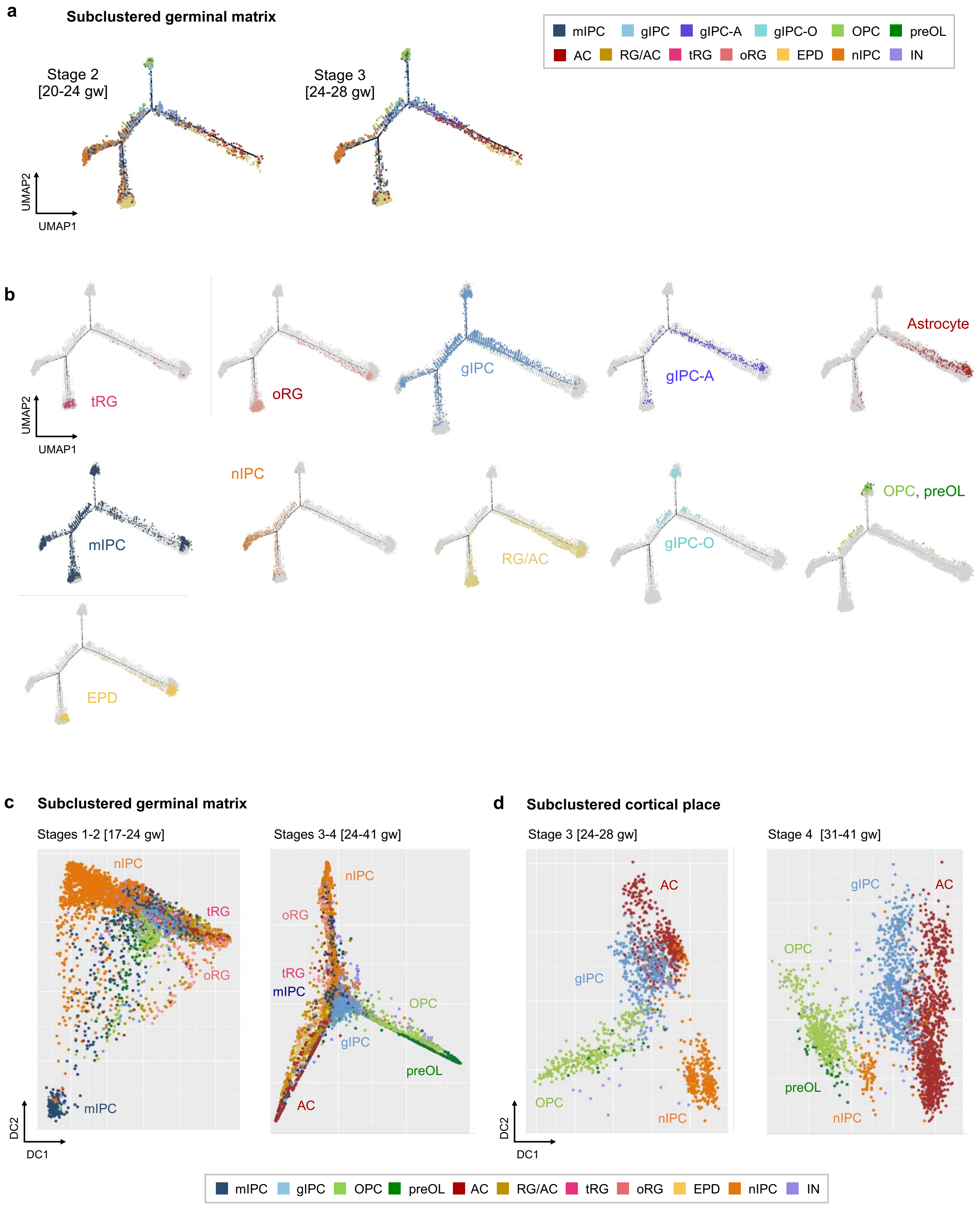

Supplementary Fig. 7 Lineage reconstruction with Monocle and Destiny

a-b. Trajectory reconstruction of GM lineages (subclustered cell type annotations) using Monocle, showing representation of all cell type annotations separated by stage (a, stage 2 and 3 shown) and colored by individual cell type (b, stages 1-4). **c-d.** Lineage analysis performed using Destiny, showing diffusion maps of subclustered GM lineages grouped into Stages 1-2 (17-24 gw) and Stages 3-4 (24-41 gw) (c) and of subclustered CP lineages split into Stage 3 and Stage 4 (d). Cells are colored by cell subtype annotations from Fig. 2**.** See also Figure 5 and 6.

Supplementary Figure 8 (relates to Figures 5 and 6)

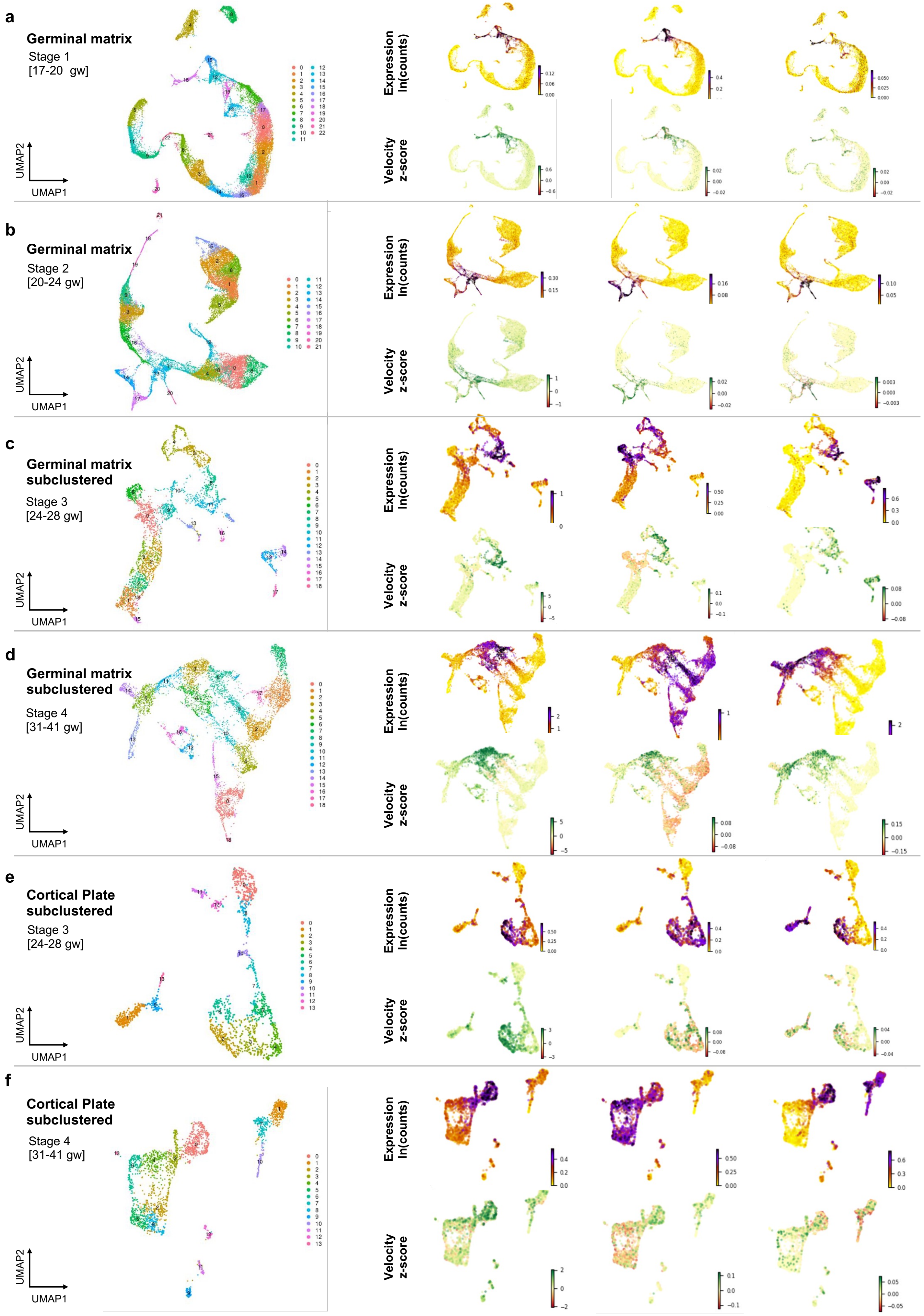

Supplementary Fig. 8 Lineage directionality analysis with scVelo. a-f. UMAP embeddings of scVelo analysis projections, colored by cluster (left) and expression and velocity plots of g-IPC (*EGFR*), astrocyte, (*FGFR3*), and OPC (*OLIG1*) lineage markers (right), for Stage 1 GM entire object (a), Stage 2 GM entire object (b), Stage 3 GM subclustered object (c), Stage 4 GM subclustered object (d), Stage 3 CP subclustered object (e), and Stage 4 CP subclustered object (f). For velocity plots green corresponds to induction while orange/red corresponds to repression, inferred from unspliced/spliced transcript reads.

Supplementary Figure 9 (relates to Figure 7)

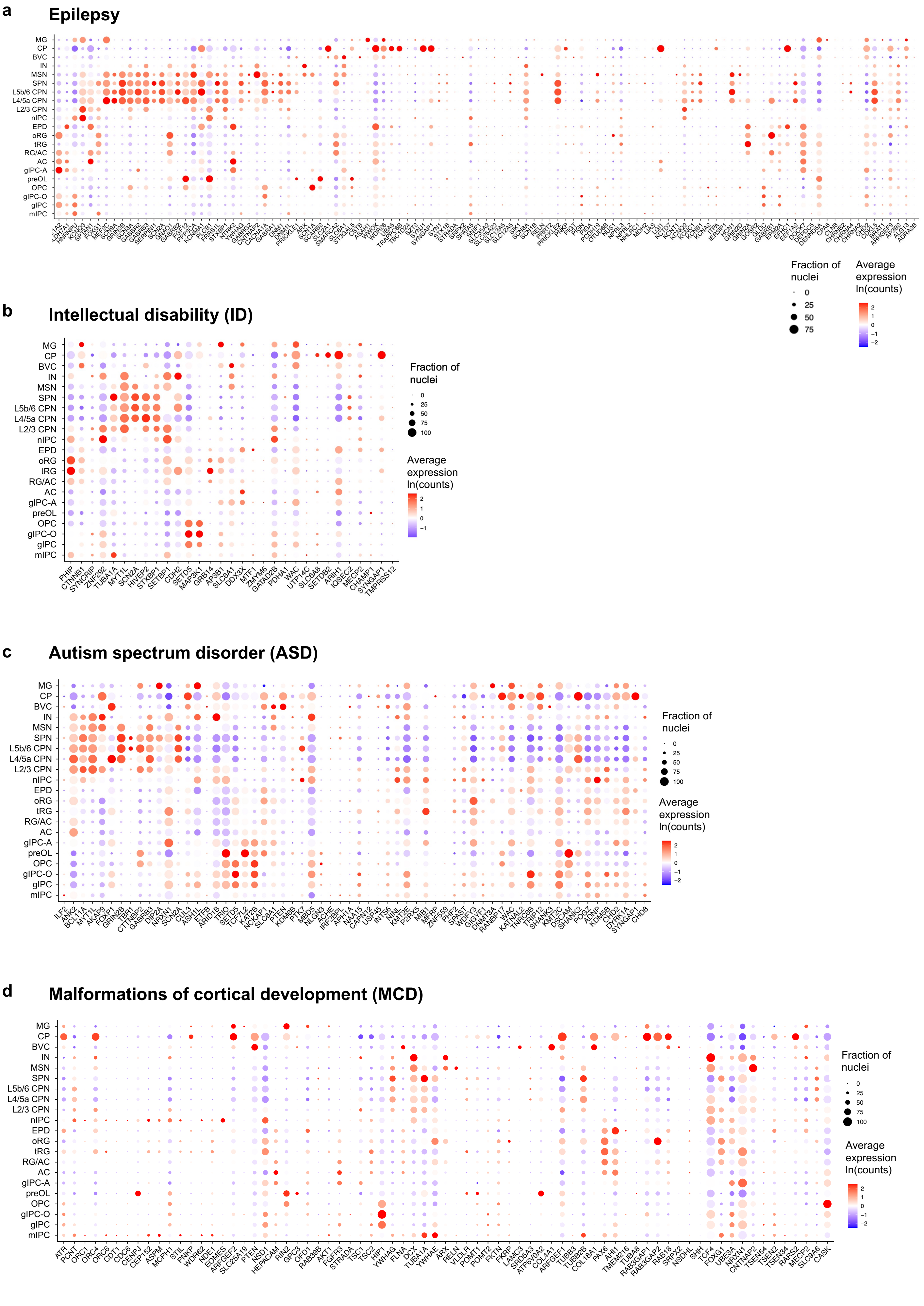

Supplementary Fig. 9 Expression of disease-associated genes in germinal matrix cell types. a-d. Dot plots of gene expression level and frequency within each annotated prenatal germinal matrix cell type for published neurodevelopmental disorder-associated genes, including epilepsy (a), intellectual disability (ID) (b), autism spectrum disorder (ASD) (c), and malformation of cortical development (MCD) (d).

Supplementary Figure 10 (relates to Figure 7)

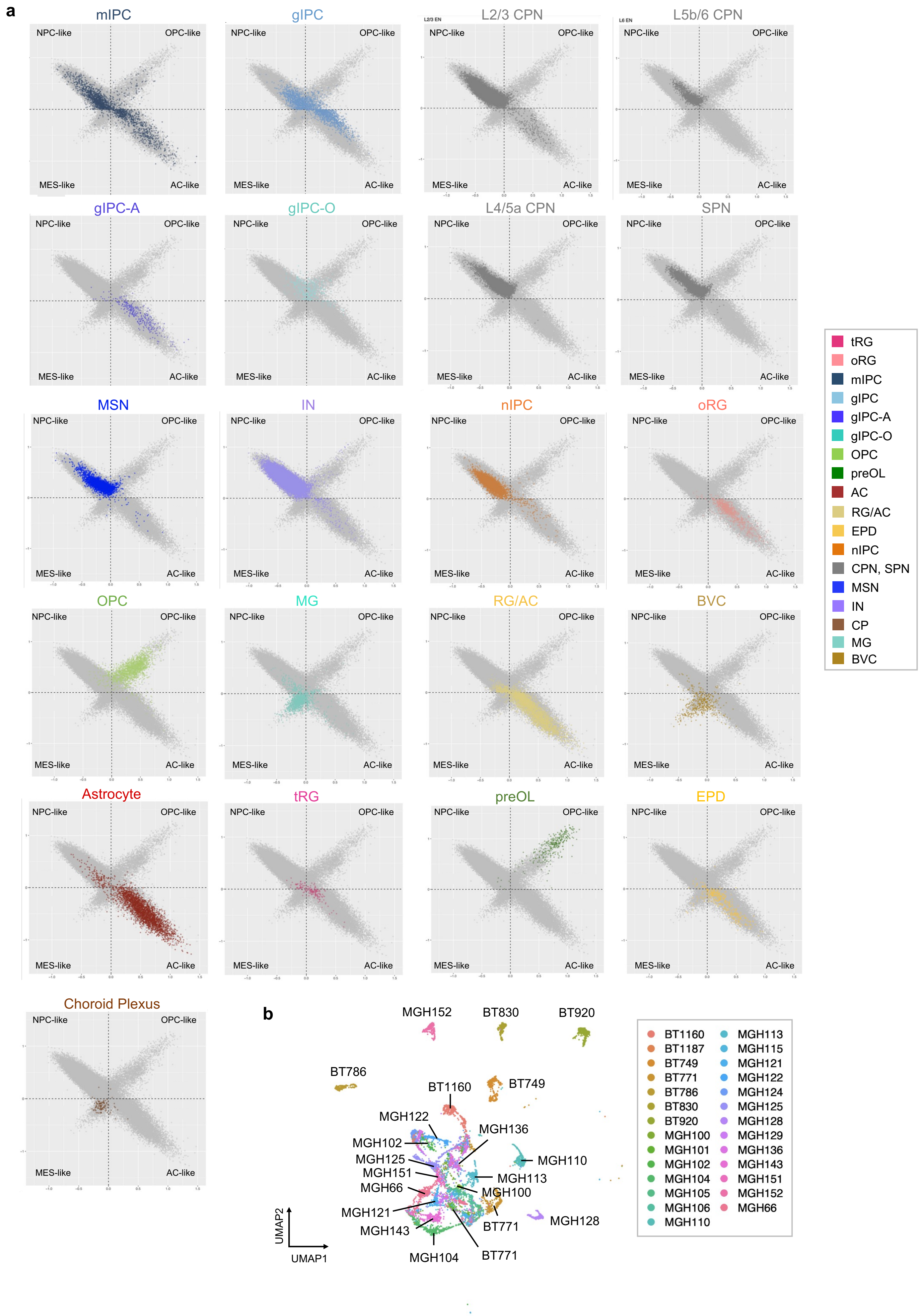

Supplementary Fig. 10 Germinal matrix cell type signatures in association with glioma

a. Scatter plot representation of Neftel et al. (2019) signature modules, used to define glioma states, and prenatal germinal matrix signatures from this study, colored by individual GM cell type, see also Figure 7c. CPN, SPN, and CRN included as part of EN. **b.** UMAP of merged Neftel et al. (2019) malignant cell dataset, colored by unique patient identity, see also Figure 7e.

Abbreviations for all supplementary Figures:

TAC = transit-amplifying cell / cycling progenitor; RG = radial glia; oRG = outer radial glia; tRG = truncated radial glia; EPD = ependymal cell; AC = astrocyte; gIPC = glial intermediate progenitor cell; OPC = oligodendrocyte progenitor cell; preOL = premyelinating / early myelinating *BCAS1*+ oligodendrocyte; OL = oligodendrocyte; nIPC = neuronal intermediate progenitor cell; mIPC = multipotent intermediate progenitor cell; UD = undefined; MSN = medium spiny neuron; EN = excitatory neuron; CPN = cortical projection neuron; SPN = subplate neuron; CRN = Cajal Retzius cell; IN = interneuron; MG = microglia; BVC = blood vessel cell; L2/3, L4, L5/6 = neocortical layers 2/3, 4, 5/6; GM or G = prenatal germinal matrix; SVZ = adult subventricular zone; CP = prenatal cortical plate or choroid plexus; CX or C = adult neocortex; gw = gestational weeks; yr = years.
