# Supplementary material for: An atlas of late prenatal human neurodevelopment resolved by single-nucleus transcriptomics
Source: Nat Commun. 2022 Dec 12;13:7671. doi: 10.1038/s41467-022-34975-2 (PMC9744747; doi:10.1038/s41467-022-34975-2)
Supplement: Supplementary file 3 — Description of Additional Supplementary Files [file 41467_2022_34975_MOESM3_ESM.pdf]

Description of Additional Supplementary Files

File Name: Supplementary Data 1
Description: Determination of prenatal gestational age in samples

File Name: Supplementary Data 2
Description: Stratification of samples by sex and distinct gestational stages

File Name: Supplementary Data 3-5
Description: Cell Ranger sequencing metrics of per stage snRNA-seq data

File Name: Supplementary Data 6
Description: Top 50 cluster-defining genes for all integrated data cluster analyses identified by two-sided Wilcoxon Rank Sum testing (p-adj. > 0.05; average log2(fold change) > 0.5 or < -0.5).

File Name: Supplementary Data 7
Description: Differential gene expression analyses of germinal matrix cell types identified by two-sided Wilcoxon Rank Sum testing (p-adj. > 0.05; average log2(fold change) > 0.5 or < -0.5).

File Name: Supplementary Data 8
Description: Gene set enrichment analysis of top 100 differential mIPC markers performed using hypergeometric testing with Benjamini-Hochberg correction (p-adj. < 0.05).

File Name: Supplementary Data 9
Description: Gene set enrichment analysis of top 100 differential gIPC markers performed using hypergeometric testing with Benjamini-Hochberg correction (p-adj. < 0.05).

File Name: Supplementary Data 10
Description: Gene set enrichment analysis of top 100 differential gIPC-O markers performed using hypergeometric testing with Benjamini-Hochberg correction (padj. < 0.05).

File Name: Supplementary Data 11
Description: Gene set enrichment analysis of top 100 differential gIPC-A markers performed using hypergeometric testing with Benjamini-Hochberg correction (p-adj. < 0.05).
