# Supplementary material for: An atlas of late prenatal human neurodevelopment resolved by single-nucleus transcriptomics
Source: Nat Commun. 2022 Dec 12;13:7671. doi: 10.1038/s41467-022-34975-2 (PMC9744747; doi:10.1038/s41467-022-34975-2)
Supplement: Supplementary file 5 — Reporting Summary [file 41467_2022_34975_MOESM5_ESM.pdf]

nature research

Corresponding author(s): Nadejda Tsankova

Last updated by author(s): Apr 30, 2021

Reporting Summary

Nature Research wishes to improve the reproducibility of the work that we publish. This form provides structure for consistency and transparency in reporting. For further information on Nature Research policies, see our Editorial Policies and the Editorial Policy Checklist.

Statistics

For all statistical analyses, confirm that the following items are present in the figure legend, table legend, main text, or Methods section.

n/a	Confirmed	
☐	☒	The exact sample size (*n*) for each experimental group/condition, given as a discrete number and unit of measurement
☐	☒	A statement on whether measurements were taken from distinct samples or whether the same sample was measured repeatedly
☐	☒	The statistical test(s) used AND whether they are one- or two-sided<br>*Only common tests should be described solely by name; describe more complex techniques in the Methods section.*
☒	☐	A description of all covariates tested
☐	☒	A description of any assumptions or corrections, such as tests of normality and adjustment for multiple comparisons
☐	☒	A full description of the statistical parameters including central tendency (e.g. means) or other basic estimates (e.g. regression coefficient) AND variation (e.g. standard deviation) or associated estimates of uncertainty (e.g. confidence intervals)
☐	☒	For null hypothesis testing, the test statistic (e.g. *F*, *t*, *r*) with confidence intervals, effect sizes, degrees of freedom and *P* value noted<br>*Give P values as exact values whenever suitable.*
☒	☐	For Bayesian analysis, information on the choice of priors and Markov chain Monte Carlo settings
☒	☐	For hierarchical and complex designs, identification of the appropriate level for tests and full reporting of outcomes
☐	☒	Estimates of effect sizes (e.g. Cohen's *d*, Pearson's *r*), indicating how they were calculated

Our web collection on statistics for biologists contains articles on many of the points above.

Software and code

Policy information about availability of computer code

Data collection	10X Genomics was used to generate single-nucleus RNA sequencing libraries.
Data analysis	Gene expression count matrices were generated with CellRanger (v3.1.5), processed and analyzed mainly using R package Seurat (v4.0.4). Seurat functions used: NormalizeData, RunPCA (2,000 variable genes), SCTransform, FindNeighbors, FindClusters, RunUMAP, CellCycleScoring, FindAllMarkers, AddModuleScore, FindTransferAnchors, Plot_cell_trajectory, AddMetaData. Regulons were analyzed using SCENIC (v1.1.2-01). Data visualization used ComplexHeatmap (v2.8.0), ggplot2 (v3.3.3), ggrepel (v0.9.1), ggvenn (v0.1.9), GraphPad Prism 9 (v9.3.1). Lineages were inferred using Monocle2 (v2.20.0), scVelo (v0.2.4), velocyto (v0.6), and Destiny (3.12.0). Doublets were removed via DoubletFinder (v2.0.3). Metascape (metascape.org, 2022 webversion) was used. Default parameters were used unless otherwise stated.

For manuscripts utilizing custom algorithms or software that are central to the research but not yet described in published literature, software must be made available to editors and reviewers. We strongly encourage code deposition in a community repository (e.g. GitHub). See the Nature Research guidelines for submitting code & software for further information.

Data

Policy information about availability of data

All manuscripts must include a data availability statement. This statement should provide the following information, where applicable:

- Accession codes, unique identifiers, or web links for publicly available datasets
- A list of figures that have associated raw data
- A description of any restrictions on data availability

Single nucleus prenatal and adult RNA-seq data generated in this study have been deposited in the GEO database under the unique, accession code GSE### and will be deposited on the UCSC browser after publication.
Previously published data used in this study for comparative studies are available in the GEO and GSA databases under accession codes GSE131928 (Neftel et al 2019, GBM); HRA000348 and GSE144462 (Yang et al 2022, Fu et al 2021; human development), and GSE161132 (Li et al 2021; mouse development).
Source data are provided with this paper (Fig. 1f, Fig. 1h, Fig. 2c, Fig. 2d, Fig. 2e, Fig. 2f, Fig. 2j, Fig. 2k, Fig. 3h, Fig. 3i, Fig. 4b, Fig. 4c, Fig. 4g, Fig. 4i, Fig. 7f, Supplemenatry Figs: 3a, 3b, 3c, 4c, 4d, 4e, 4f, 4g, 4h, 4i, 5a, 5b, 6a, 6b, 6c, 6d, 6e).

Field-specific reporting

Please select the one below that is the best fit for your research. If you are not sure, read the appropriate sections before making your selection.

☒ Life sciences ☐ Behavioural & social sciences ☐ Ecological, evolutionary & environmental sciences

For a reference copy of the document with all sections, see nature.com/documents/nr-reporting-summary-flat.pdf

Life sciences study design

All studies must disclose on these points even when the disclosure is negative.

Sample size: This study used sample size of n=3-5 per developmental time point, 20,000 nuclei on average, which we determined to be statistically different using one-way ANOVA. We aimed for high temporal resolution in our study (4 developmental stages) and chose n=3 as the minimal sample size allowing for statistical testing using Student t-test. Statistical tests relying on nuclei numbers or Spearman correlation were also used.

Data exclusions: We excluded low-quality nuclei (< 400 genes, < 1,000 UMI counts), doublets, and nuclei with mitochondrial content greater than 15%.

Replication: We successfully validated newly discovered markers in situ in two different regions, in at least 3 independent experiments. We used 3 independent computational tools to infer lineage trajectories (scVelo, Monocle2, and Diffusion Maps). Similar results were seen with all computational inferences when performed using 15% mitochondrial filter (revisions) and 25% mitochondrial filter (orginial submission).

Randomization: We used randomization in our scVelo trajectory analysis, which did not inlcude a starting lineage point.

Blinding: We were blinded by gestational age but not by anatomical region in our data integration and cell type annotation based on clustering. Our annotation methods included unbiased clustering in combination with cannonical marker expression to discover new prenatal cell types.

Reporting for specific materials, systems and methods

We require information from authors about some types of materials, experimental systems and methods used in many studies. Here, indicate whether each material, system or method listed is relevant to your study. If you are not sure if a list item applies to your research, read the appropriate section before selecting a response.

Materials & experimental systems

n/a	Involved in the study	
☐	☒	Antibodies
☒	☐	Eukaryotic cell lines
☒	☐	Palaeontology and archaeology
☒	☐	Animals and other organisms
☐	☒	Human research participants
☒	☐	Clinical data
☒	☐	Dual use research of concern

Methods

n/a	Involved in the study	
☒	☐	ChIP-seq
☒	☐	Flow cytometry
☒	☐	MRI-based neuroimaging

Antibodies

Antibodies used:
Goat anti-EGFR, 1:50, R&D Systems AF231, lot#AUC1219011
Mouse anti-EGFR, 1:50, Abcam ab218383, lot#GR3290628-5
Rabbit anti-SOX9, 1:200, Abcam ab185966, lot#GR3241181-4
Rabbit anti-OLIG2, 1:50, Millipore AB9610, lot#3524758 Goat anti-OLIG2, 1:200, R&D Systems AF2418, lot#UPA0921091
Rabbit anti-Ki67, 1:50, Invitrogen PA1-38032, lot#UI2842372A
Rabbit anti-ZEB1, 1:500, Invitrogen PA5-82982, lot#XF3610564A
Donkey anti-rabbit Alexa Fluor 488, Jackson ImmunoResearch Labs 711545152, lot#147626
Donkey anti-goat Alexa Fluor 594, Jackson ImmunoResearch Labs NC0281835, lot#125283

Validation:
EGFR: Validation on human adult tissue for IHC-P provided on manufacturer's website and on our GBM tissue.
SOX9: Validation on human prenatal tissue (9 gestational weeks) and cancer tissue for IHC-P provided on manufacturer's website.
OLIG2: Validation on human glioblastoma tissue for IHC-P provided on manufacturer's website and on our GBM tissue.
KI67: Validation on human cancer tissue for IHC-P provided on manufacturer's website and on our GBM tissue.
ZEB1: validation on human cortex, glioma, and liver tissue for IHC-P provided on manufacturer's website.

Human research participants

Policy information about studies involving human research participants

Population characteristics: 15 postmortem prenatal (17-41 gestational weeks, 7F, 8M) and 3 postmortem adult (28-53 years, 2F, 1M) de-identified autopsy brain samples without pathological abnormalities.

Recruitment: All autopsies were done with written consent from the legal next-of-kin. Tissue collection was performed de-identified in accordance with the policies and regulation at the Icahn School of Medicine at Mount Sinai (ISMMS) and its Institutional Review Board. Only autopsy tissue secondary to unexpected and involuntary pregnancy loss was collected.

Ethics oversight

The Icahn School of Medicine Institutional Review Board considers autopsies as non-human subjects. Utilization of de-identified autopsy tissue was determined as non-human research by the ISMMS IRB, and approved under exemption HS#14-01007, in accordance with ethical conduct of research and compliance with fedearal, state, and institutional regulations.

.

e approval of the study protocol must also be provided in the manuscript.
